# Global Research Status of Multiple Organ Dysfunction Syndrome During 2001–2021: A 20-Year Bibliometric Analysis

**DOI:** 10.3389/fmed.2022.814381

**Published:** 2022-03-04

**Authors:** Peng-yue Zhao, Yun Xia, Zheng-bo Tao, Song-yan Li, Zhi Mao, Xing-peng Yang, Ren-qi Yao, Xiao-hui Du

**Affiliations:** ^1^Department of General Surgery, First Medical Center of Chinese PLA General Hospital, Beijing, China; ^2^Translational Medicine Research Center, Medical Innovation Research Division and Fourth Medical Center of the Chinese PLA General Hospital, Beijing, China; ^3^Department of Anesthesiology, Zhongnan Hospital of Wuhan University, Wuhan, China; ^4^Department of Orthopedics, Changzheng Hospital, Naval Medical University, Shanghai, China; ^5^Department of Critical Care Medicine, First Medical Center of Chinese PLA General Hospital, Beijing, China; ^6^Department of Burn Surgery, The First Affiliated Hospital of Naval Medical University, Shanghai, China

**Keywords:** MODS, bibliometric analysis, publication, keywords, research hotspots

## Abstract

**Background:**

Multiple Organ Dysfunction Syndrome (MODS) is a major cause of high morbidity and mortality among patients in intensive care units (ICU). Although numerous basic and clinical researches on MODS have been conducted, there is still a long way to go to prevent patients from entering this stage. To our knowledge, no bibliometric analyses of MODS have been reported, this study, therefore, was conducted to reveal MODS research status and trends during 2001–2021.

**Methods:**

All relevant literature covering MODS during 2001–2021 were extracted from Web of Science. An online analysis platform of literature metrology was used to analyze the publication trends. VOSviewer software was used to collect and analyze the keywords and research hotspots related to MODS.

**Results:**

As of July 31, 2021, a total of 994 MODS-related articles from 2001 to 2021 were identified. The United States accounted for the largest number of publications (31.1%), followed by China and Germany, with 186 and 75 publications, respectively. Among all the institutions, the University of Pittsburgh published the most papers related to MODS (21). *Critical Care Medicine* published the most papers in this field (106). Professor Moore EE, who had the most citation frequency (1847), made great achievements in MODS research. Moreover, analysis of the keywords identified three MODS research hotspot clusters: “mechanism-related research,” “clinical research,” and “diagnostic research.”

**Conclusions:**

The United States maintained a top position worldwide and made the most outstanding contribution in the MODS field. In terms of publication, China was next only to the United States, but there was a disproportion between the quantity of publications and citation frequency. The institution University of Pittsburgh and journal *Critical Care Medicine* represent the highest level of research in this field. During the 20 years from 2001 to 2021, basic MODS research has been in-depth yet progressed relatively slowly recently, but the outbreak of COVID-19 has to some extent set off an upsurge of clinical research in MODS field.

## Introduction

The high fatality rate of coronavirus disease (COVID-19) has posed a serious challenge to global health and epidemic prevention, and the latest research shows that this is closely related to the later progression of severe COVID-19 patients to Multiple Organ Dysfunction Syndrome (MODS) (1, 2). MODS is defined as the acute and potentially reversible dysfunction of two or more organs triggered by multiple clinical or non-clinical factors. The concept of MODS was first proposed in 1992, which was previously known as multiple organ failure (MOF) (3). Given that MOF could only be described statically, without showing a continuous process of multiple organ dysfunction, the concept of MODS came into being and gradually replaced MOF (4). The organ or system most easily affected by MODS successively include lung, cardiovascular system, liver, kidney, blood system, gastrointestinal tract, and central nervous system. The mortality in MODS patients increases with the number of organs involved. When only two organs become dysfunctional, the mortality is about 30%; when 3 or 4 organs are affected, the mortality will rise to 50–70% (5, 6).

Two primary causes of MODS are infectious and non-infectious factors, especially the former. Specifically, common causes, in addition to the most common cause of sepsis, include trauma, burn, surgery, shock, and so on (5, 7). Although an underlying pathophysiology for MODS remains elusive, global perfusion deficits (8), widespread endothelial damage (9), mitochondrial dysfunction/hibernation and associated energy deficit (10), intestinal bacterial product translocation (11), and apoptosis (12) have been implicated. These pathological mechanisms may aggravate the dysfunction of various organs (13–16). In recent years, mitochondrial dysfunction and abnormalities in the quality control methods of immune cells (the aggravation of apoptosis, the increase of pyrolysis, the dysfunction of autophagy and so on) have gradually attracted the attention of relevant researchers (17, 18). However, in the field of MODS, both basic research and clinical research are progressing slowly. It is no exaggeration to say that there has been no substantial breakthrough in the pathophysiology of MODS.

If we can summarize the current research status of MODS based on the available literature and analyze the current research difficulties and future research hotspots, it is expected to provide reference and develop new ideas for MODS related researchers. Bibliometrics is the tool to achieve the above goals. Bibliometrics refers to the cross-science of using mathematical and statistical methods to quantitatively and qualitatively analyze all knowledge carriers including books, periodicals and other publications (19). It is a comprehensive knowledge system that integrates mathematics, statistics, and philology, with a special focus on quantification. Bibliometric analysis can not only compare the contributions of different countries, institutions, journals, and scholars, but also describe a specific research field and predict the specific trends and future research hotspots, thus making important contributions to the prevention and treatment of diseases (20, 21). Although there are many bibliometric analyses on sepsis, the bibliometric research on MODS has not yet been reported, which means that there is a lack of comprehensive analysis of MODS and prediction of its research hotspots.

This research aims to analyze the content of MODS-related research literature based on Web of Science (WOS). We intend to use bibliometric methods to conduct a comprehensive analysis of the research status of MODS in the past 20 years, reveal research trends in this field and predict possible future research hotspots, in the hope of providing reference for the clinical treatment and scientific research of MODS.

## Materials and Methods

### Data Sources and Search Strategies

**Figure 1:**
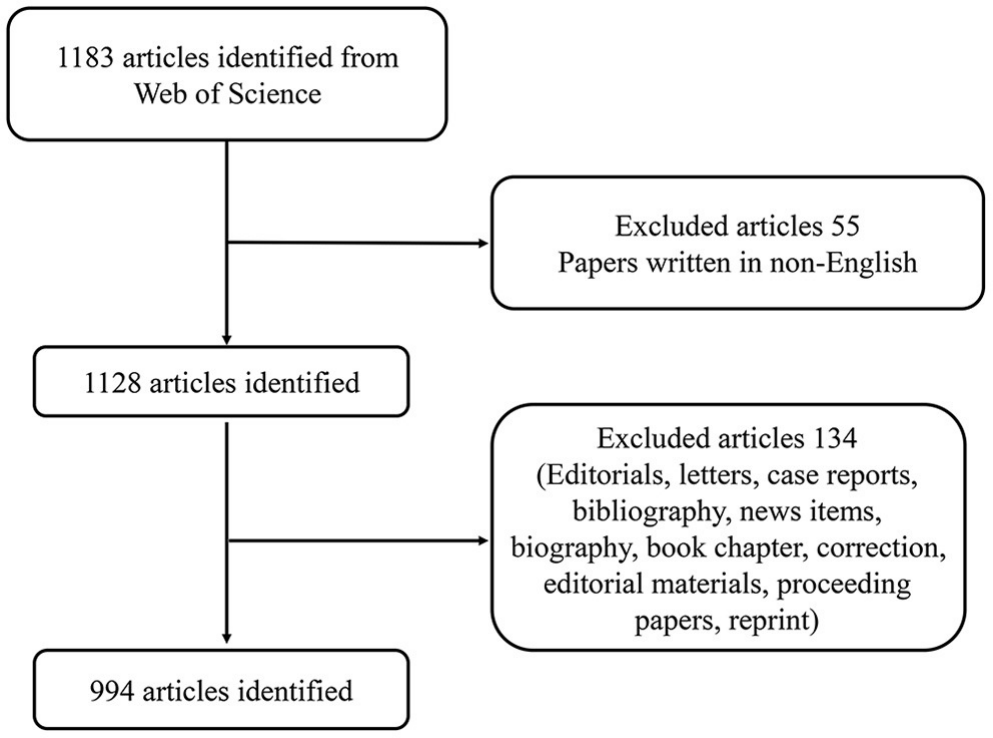
Flow diagram of literature selection and screening in this study.

The science citation index extension (SCIE) of Thomson Reuters Science Network is the most suitable database for bibliometric analysis. We used the WOS database to perform a comprehensive online search for literature published from 2001 to 2021. The article types were limited to original articles and reviews. The search strategy was as follows: TI = (Multiple Organ Dysfunction Syndrome) OR TI = (MODS) OR TI = (Organ Failure, Multiple) OR TI = (Multiple Organ Failures) OR TI = (Organ Dysfunction Syndrome, Multiple) AND Language = English. To avoid the prejudice caused by frequent updates of the database, all literature searches and data collection were completed within a single day on July 31, 2021. In addition, all data were obtained from public databases and did not involve any human subjects. For this reason, there was no need for informed consent. The detailed process of literature selection and screening is shown in Figure 1.

### Data Collection

Two reviewers (ZPY and XY) independently conducted primary search and extracted data from all qualified literature. The extracted data from WOS included titles, key words, publication dates, countries and regions, authors, institutions, journals, total number of citations, and H-index. Microsoft Excel 2016 (Redmond, Washington, USA), VOSviewer software (Leiden University, Leiden, Netherlands) and online platform of bibliometrics (http://bibliometric.com/) were used for qualitative and quantitative analyses.

### Bibliometric Analysis

All publication characteristics of qualified documents in WOS, including country, institution, journal, author, H-index, etc., were recorded and described in detail. By examining the latest issue of JCR (Journal Citation Reports), we obtained the latest impact factor (IF) of the relevant journals, an important indicator to evaluate the academic influence of research (22). The H-index is an index to quantify an individual's scientific research output, which is defined as a scholar or a country has published h papers, and each paper have at least h citations (23). H-index can be obtained from WOS, reflecting the academic influence of scholars or countries/regions. In this study, we analyzed the number of publications and growth trends in different countries/regions annually using the online analysis platform of bibliometrics. VOSviewer software was applied to visualize keyword networks extracted from MODS research, thereby categorizing keywords into different clusters based on co-occurrence analysis. Moreover, it colored each keyword in line with their emerging time, for which we applied the definition of Average Year of Appearance (AAY) to quantify the relative novelty of keywords.

## Results

### Global Growth Trends of Publications

**Figure 2:**
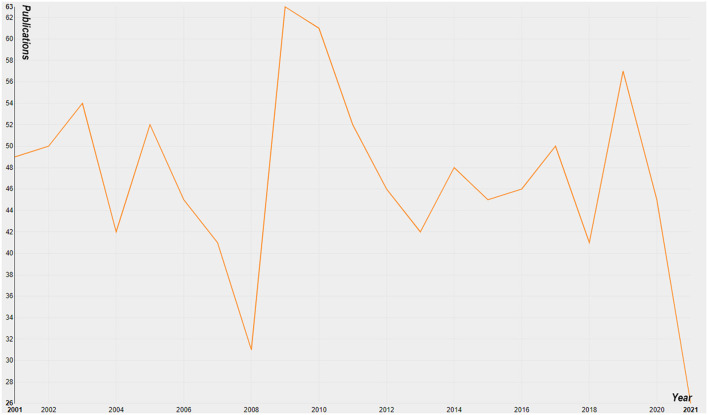
The number of annual publications in MODS from 2001 to 2021.

A total of 994 articles published from 2001 to 2021 met our inclusion criteria (Figure 1). As shown in Figure 2, the global trend of published literature on MODS research was plotted. The years 2009 (63, 6.3%) and 2019 (57, 5.7%) were the 2 years with the highest volume of MODS-related publications. We also investigated the cumulative number of publications globally and found that the total publication of MODS-related literature has shown a trend of steady growth.

**Figure 3:**
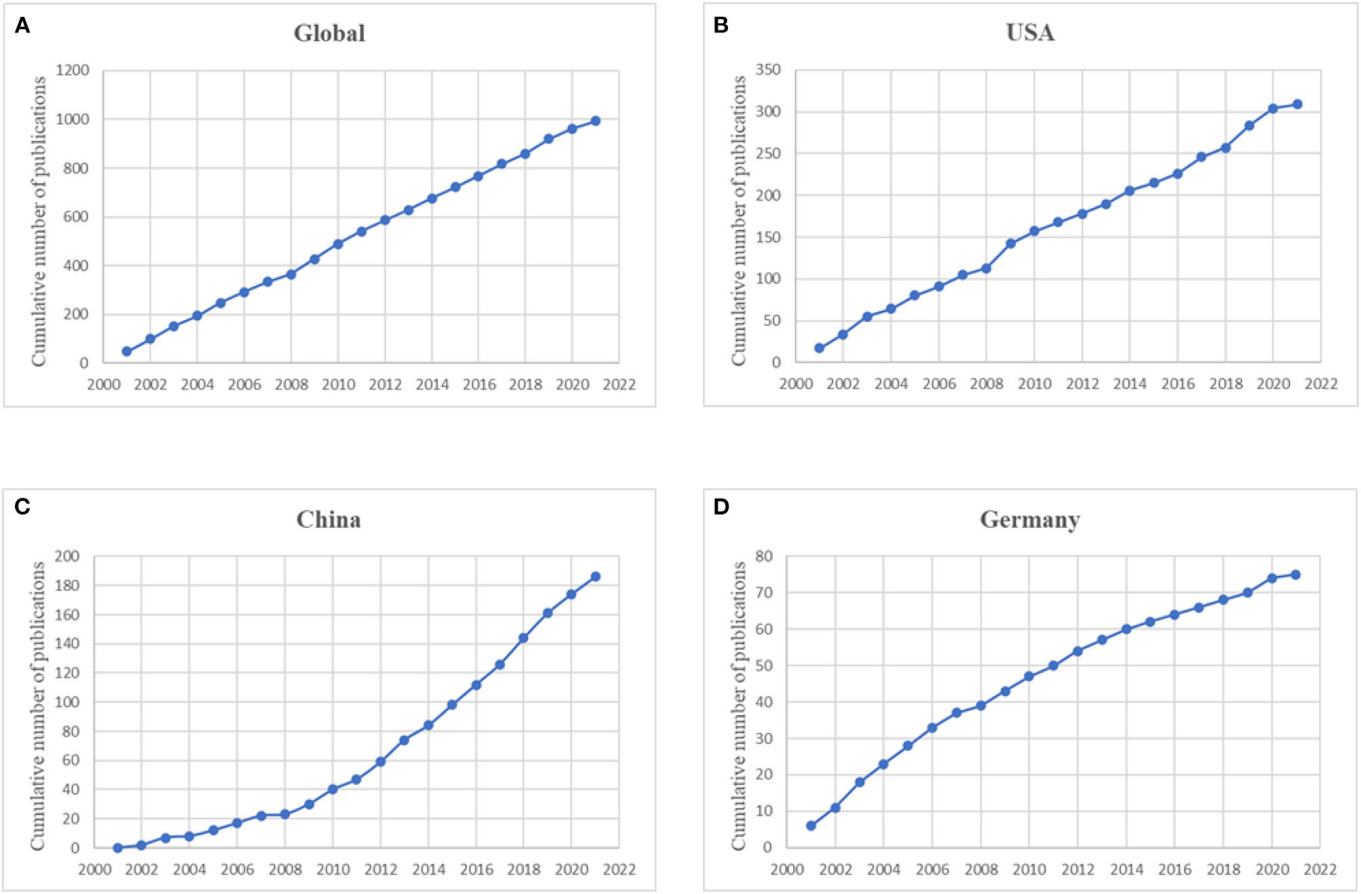
The model fitting curves of growth trends of publications related to MODS. **(A)** Global; **(B)** the United States; **(C)** China; **(D)** Germany.

Meanwhile, we selected the top three countries with the highest volume of publications (the United States, China, and Germany), and compared their publication trends. The results showed that the trend of the United States was basically identical with that of the world, whereas publication in China displayed a relatively faster growth curve. Conversely, the Germany's growth trend showed a gradual downward curve (Figure 3).

### Contribution of Countries to Global Publications

**Figure 4:**
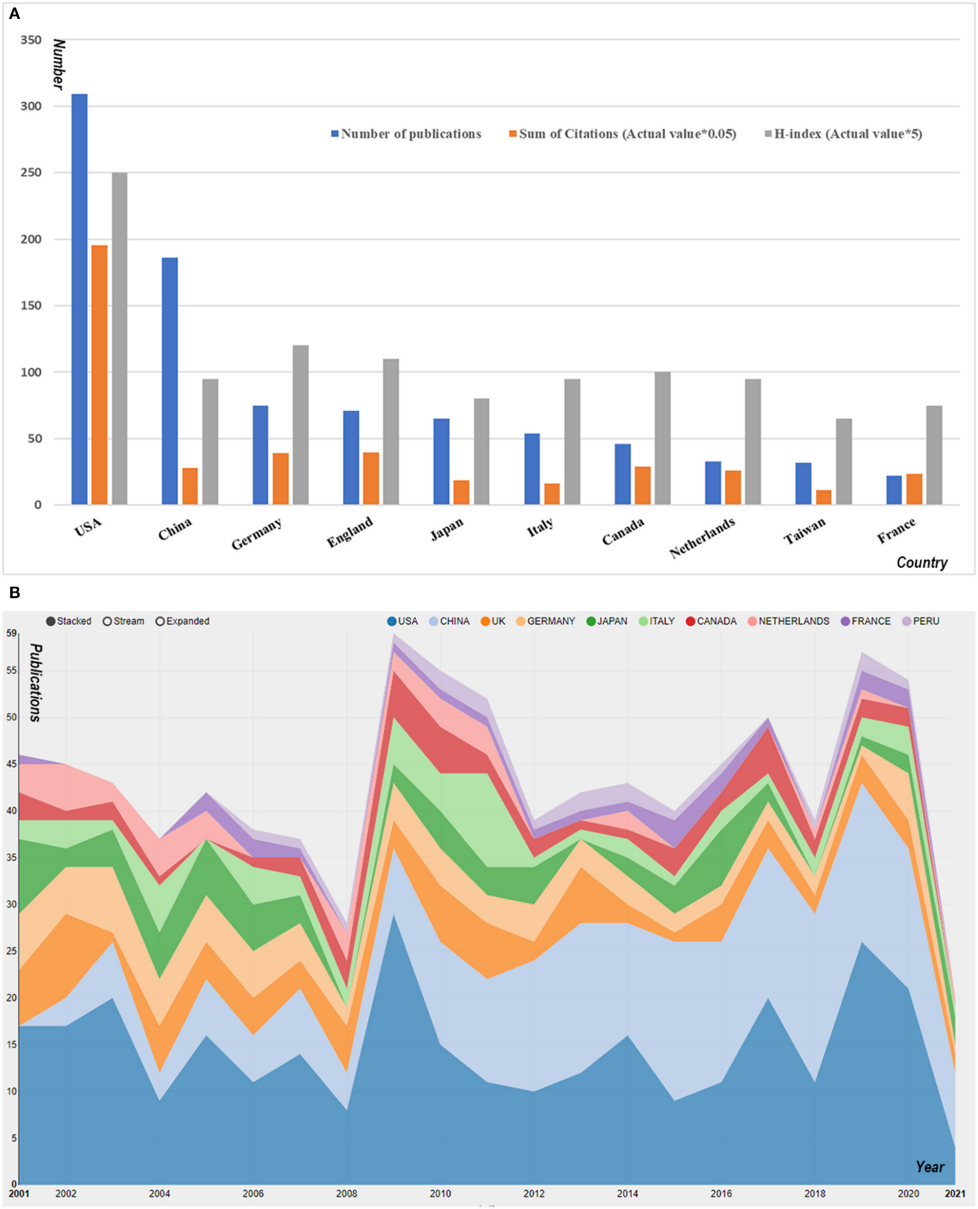
The contributions of different countries/regions to the research field concerning MODS. **(A)** The number of publications, citation frequency (×0.05), and H-index (×5) in the top 10 countries or regions. **(B)** The growth trends of the top 10 countries/regions in MODS from 2001 to 2021.

The United States ranked first regarding the number of publications (309, 31.1%), followed by China (186, 18.7%), Germany (75, 7.5%), England (71, 7.1%) and Japan (65, 6.5%). The detailed data of the top 10 countries/regions in terms of number of publications are presented in Figure 4A. The results of the cited frequency report from the WOS database showed that the 994 articles related to MODS were cited 21,363 times since 2001 (20,320 times without self-citation). The average citation frequency was 21.38 times per literature, and the H-index was 75. The citation frequency of the United States (9,773 times, 9,504 times without self-citation) accounted for 45.7% of the total. The average citations per literature was 31.63 times, with an H-index of 50. Publications by Germany were cited for 1,948 times (1,908 times without self-citation), with an H-index of 24, ranking second among all countries. China's publication number was second only to the United States in this field, but its citation frequency was merely 1,395 times with an H-index of 19, ranking 5th and 7th, respectively. What is worth mentioning is that the publications on MODS research conducted by Chinese scholars increased sharply since 2010. In 2012, China's annual publication volume surpassed the United States for the first time. Since then, the annual volume of MODS-related publications in China and the United States were comparable (Figure 4B).

**Figure 5:**
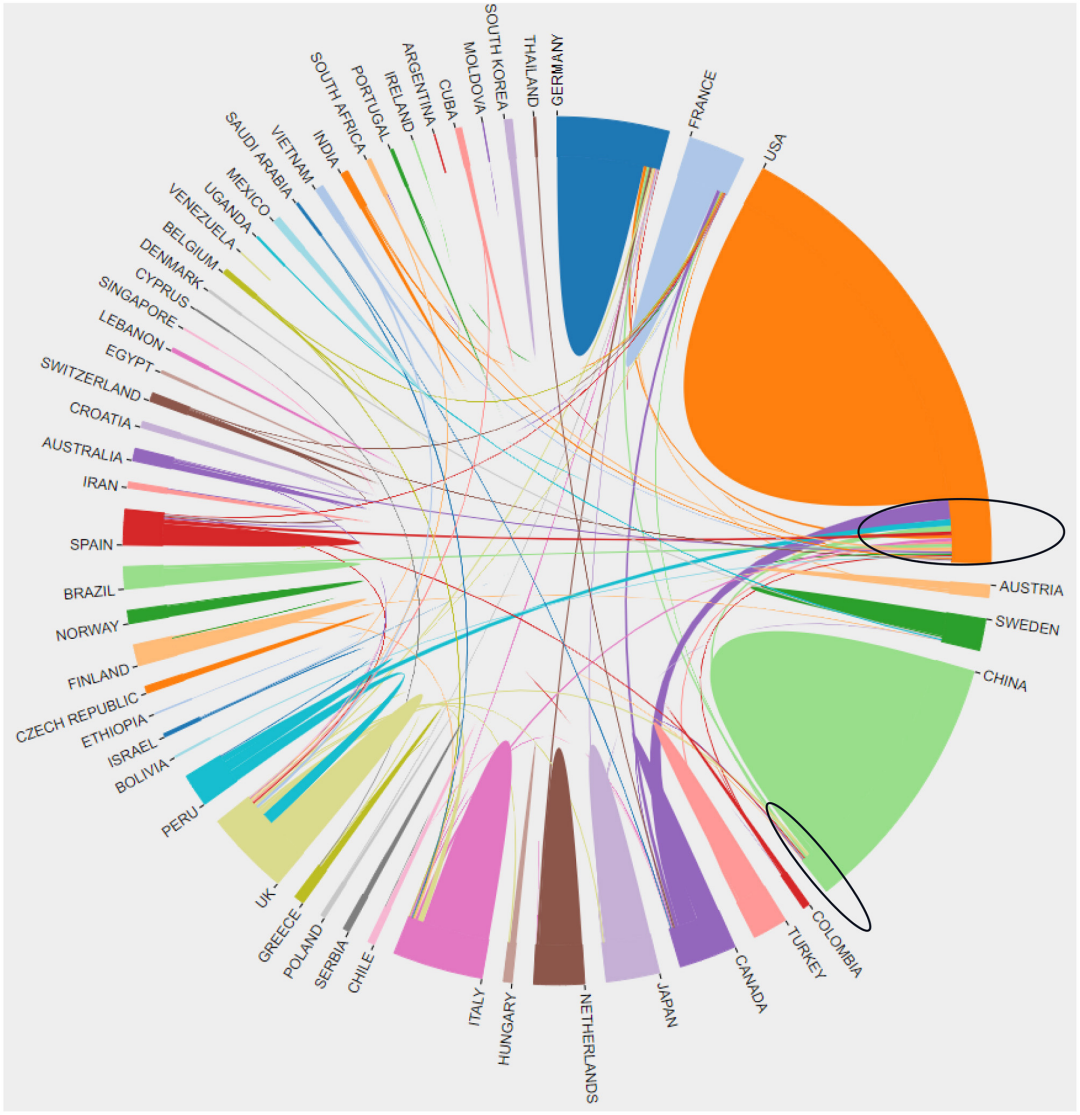
The cooperation of countries/regions in MODS from 2001 to 2021.

To gain insight into the collaborative level between countries and regions worldwide, we mapped the cooperation of countries/regions using VOSviewer. Although the United States and China were the two leading countries with the greatest number of publications, the former had closer international cooperation compared to the latter, as evidenced by the central position of the U.S. among co-occurrence network (Figure 5).

Meanwhile, we conducted an analysis on publications in each country/region upon time course. The results showed that the papers in Europe, the United States, and other countries were predominantly published 10 years ago, while China published numerous MODS-related literature in the past 5 years (Supplementary Figure 1).

### Contribution of Institutions to Global Research on MODS

**Figure 6:**
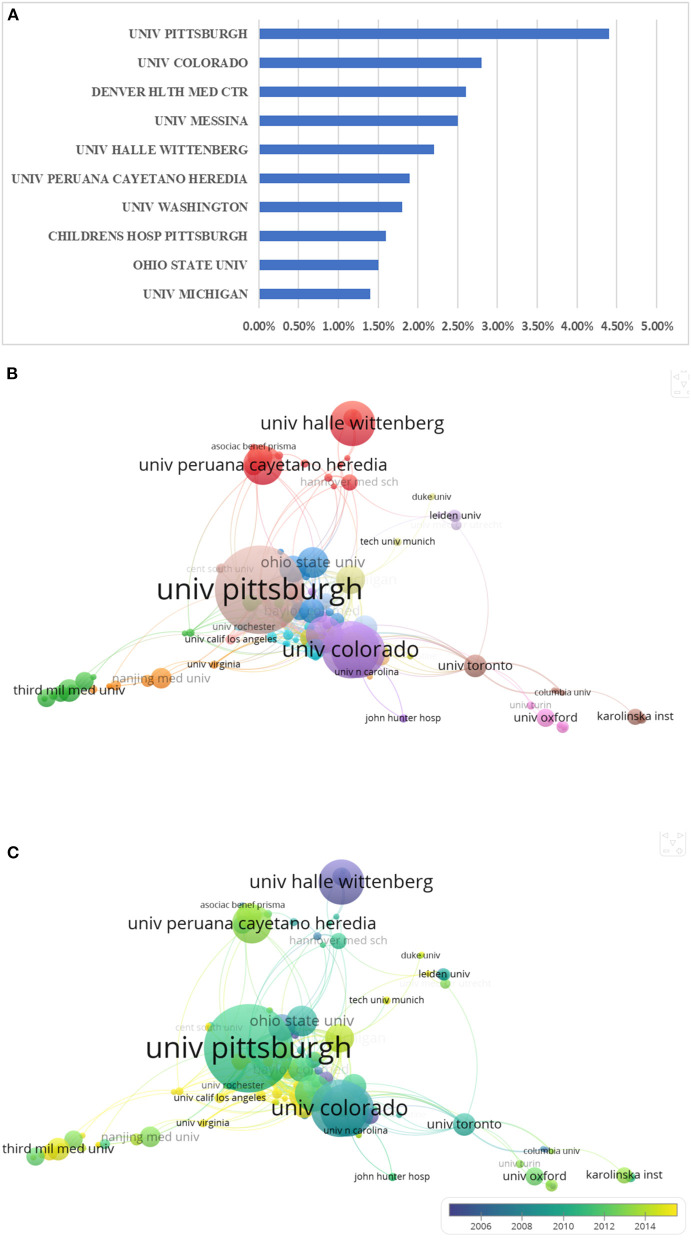
The contributions of different institutions focusing on MODS. **(A)** Distribution of top 10 institutions focusing on MODS; **(B)** The cooperation of institutions in MODS, the circle with a large size represented the institution that published more articles; **(C)** Distribution of institutions was presented according to the appearance for the average time.

Globally, the University of Pittsburgh was the institution that published the highest number of MODS-related literature in the past 20 years (44, 4.4%). Among the top 10 institutions in this field, 7 of them were from the United States, and the other 3 were the University of Messina in Italy, the University of Halle-Wittenberg in Germany, and the University of Cayetano in Peru (Figure 6A). Furthermore, we analyzed the cooperative relationships of major institutions. The University of Pittsburgh and the University of Colorado in the United States located at the center of the map, indicating their close international cooperation. Besides, it was noteworthy to integrate and analyze the publications by major institutions over time. The results revealed that the top 10 institutions published MODS-related literature primarily 10 years ago, whereas the Third Military Medical University from China published several novel articles in recent 5 years (Figures 6B,C).

### Journals and Authors Publishing Research on MODS

**Figure 7:**
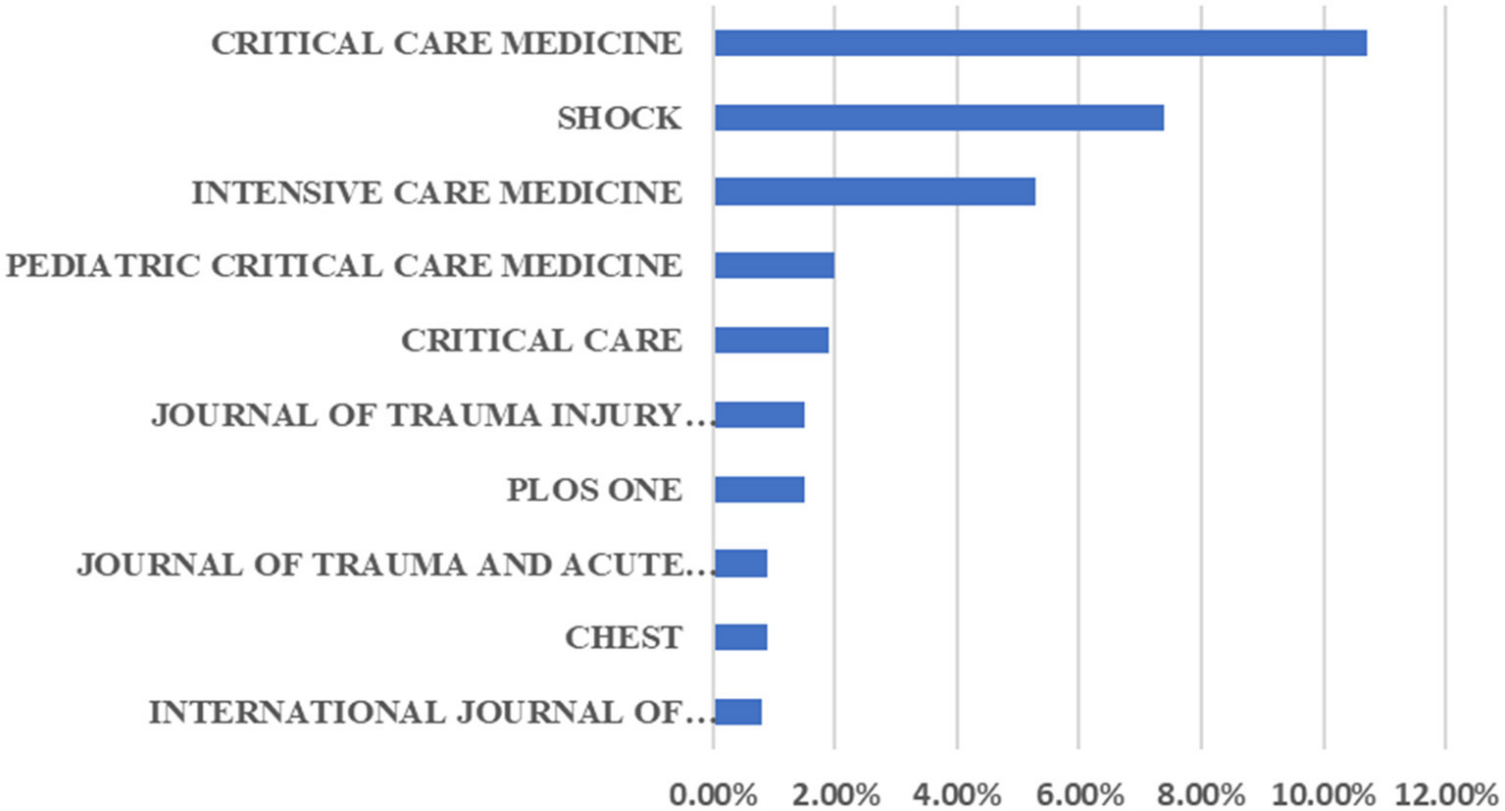
The top 10 journals publishing research on MODS.

A total of 328 articles related to MODS were included in the top 10 journals with respect to the number of publications, accounting for approximately one-third (33%) of the overall publications. *Critical Care Medicine* (106), *Shock* (74) and *Intensive Care Medicine* (53) ranked the top three, with related literature accounting for 23.4% of all publications (Figure 7). Moreover, we listed the top 10 most influential MODS-related works based on the citation frequency, among which the most cited one entitled “Immunosuppression in Patients Who Die of Sepsis and Multiple Organ Failure” was conducted by Hotchkiss et al. and published on *Journal of the American Medical Association (JAMA)* in 2011 (24). The total citations and average annual citations of this paper were as high as 884 and 80.36, respectively. As to the top 10 papers, *Lancet* and *the Journal of Trauma* published two articles each, whereas the rest of articles were issued on other journals (Table 1).

**Table 1:** Top 10 high-cited papers related to MODS.

**Title**	**Corresponding authors**	**Journal**	**IF**	**Publication year**	**Total citations**	**Average per year**
Immunosuppression in Patients Who Die of Sepsis and Multiple Organ Failure	Hotchkiss, Richard S.	JAMA	56.272	2011	884	80.36
The role of the endothelium in severe sepsis and multiple organ dysfunction syndrome	Aird, W. C.	BLOOD	22.113	2003	763	40.16
Neutrophils in development of multiple organ failure in sepsis	Treacher, D. F.	LANCET	79.320	2006	412	25.75
Post-injury multiple organ failure: The role of the gut	Moore, F. A.	SHOCK	3.450	2001	389	18.52
Continuous venovenous hemodiafiltration vs. intermittent hemodialysis for acute renal failure in patients with multiple-organ dysfunction syndrome: a multicenter randomized trial	Dhainaut, Jean-Francois	LANCET	79.320	2006	376	23.5
Inflammation, coagulopathy, and the pathogenesis of multiple organ dysfunction syndrome	Marshall, J. C.	CRITICAL CARE MEDICINE	7.590	2001	284	13.52
Both primary and secondary abdominal compartment syndrome can be predicted early and are harbingers of multiple organ failure	Moore, F. A.	THE JOURNAL OF TRAUMA	NF	2003	271	14.26
Fresh Frozen Plasma Is Independently Associated With a Higher Risk of Multiple Organ Failure and Acute Respiratory Distress Syndrome	Peitzman, Andrew B.	THE JOURNAL OF TRAUMA	NF	2009	238	18.31
Microparticles from patients with multiple organ dysfunction syndrome and sepsis support coagulation through multiple mechanisms	Sturk, A.	THROMBOSIS AND HAEMOSTASIS	5.243	2001	215	10.24
The cytokine storm and factors determining the sequence and severity of organ dysfunction in multiple organ dysfunction syndrome	Ma, Sui	AMERICAN JOURNAL OF EMERGENCY MEDICINE	2.462	2008	213	15.21

*IF, impact factor; NF, Not found*.

There were 202 articles published by the top 10 authors, accounting for 20.3% of all MODS-related literature. Moore EE from the University of Pittsburgh published 30 MODS-related articles, which was cited 1,847 times, ranking first in both categories among global scholars. Italian professor Cuzzocrea S and American professor Carcillo JA published 27 and 24 articles on MODS research, ranking second and third, respectively. As shown in Table 2, among the top 10 most productive authors, four of them were from the United States, four came from Italy, and two were German authors. We further conducted the collaborative level analysis among the top-ranking authors using VOSviewer (Supplementary Figure 2).

**Table 2:** Top 10 authors with most publications in research scope of MODS.

**Author**	**Country**	**Affiliation**	**No. of publications**	**No. of citations**	**Average**	**H-Index**
Moore EE.	USA	University of Pittsburgh	30	1,847	61.57	21
Cuzzocrea S.	Italy	University of Messina	27	458	16.96	14
Carcillo JA.	USA	University of Pittsburgh	24	1,032	43	14
Mazzon E.	Italy	University of Messina	21	335	15.95	11
Werdan K.	Germany	Martin Luther University	21	543	25.86	10
Di Paola R.	Italy	University of Messina	18	220	12.22	11
Hall MW.	USA	University of Pittsburgh	17	566	33.29	7
Sauaia A.	USA	Denver Health Medical Center	16	731	45.69	10
Schmidt H.	Germany	Martin Luther University	14	467	33.36	7
Thiemermann. C	Italy	University of Messina	14	204	14.57	6

### Analysis of Keywords and Research Hotspots on MODS

**Figure 8:**
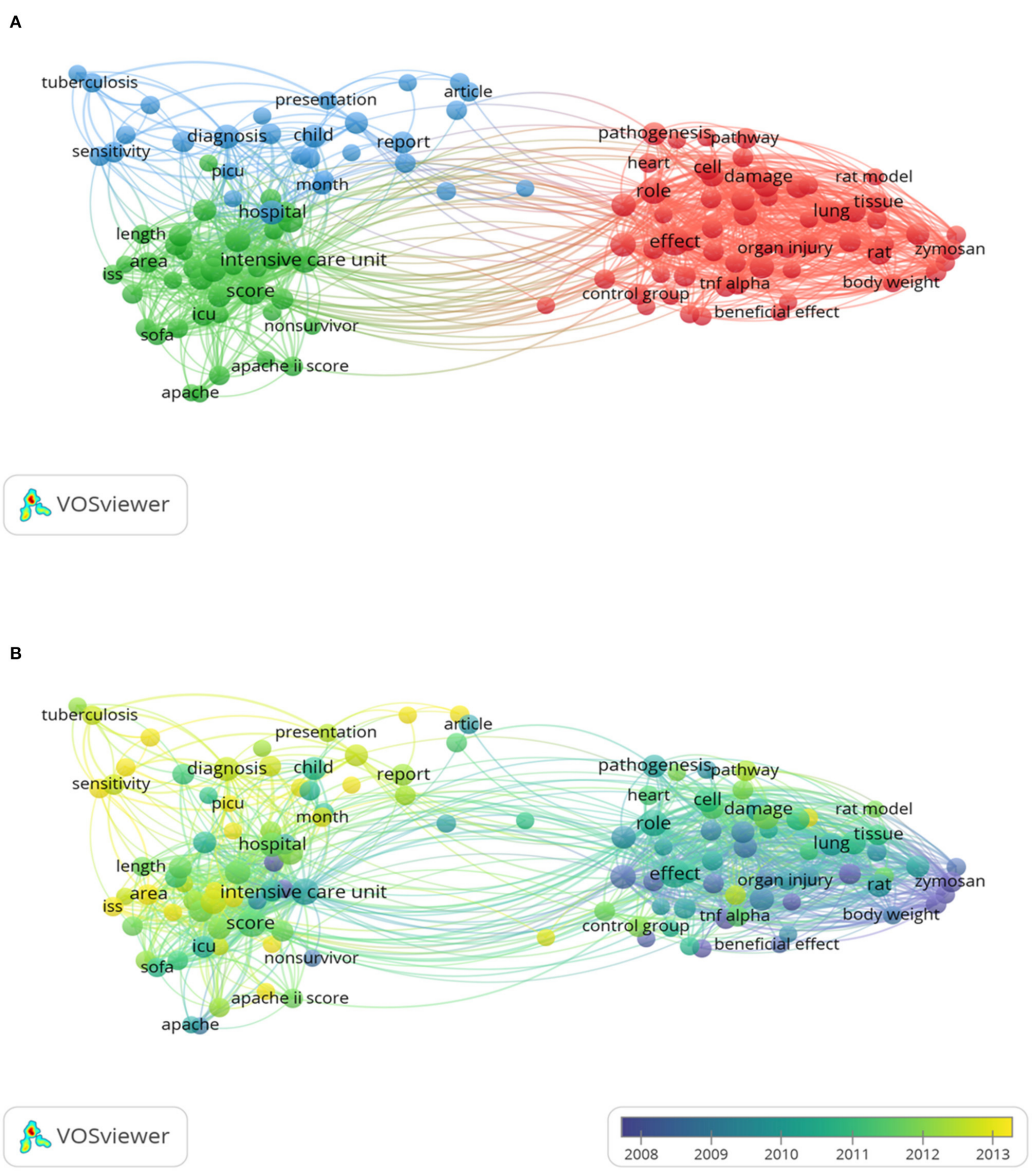
The analysis of keywords in publications of MODS. **(A)** Mapping of the keywords in the area of MODS. The words were divided into 3 cluster in accordance with different colors generated by default: mechanism research (right in red), clinical research (left in green) and diagnostics and laboratory research (up in blue). The circle with a large size represented the keywords that appeared at a high frequency; **(B)** Distribution of keywords was presented according to the appearance for the average time. The blue color represented early appearance and yellow color recent appearance. Two keywords co-occurred if they both occurred on the same line in the corpus file. The smaller the distance between two keywords, the larger the number of co-occurrences of the keywords.

We extracted keywords from the title and abstract of 994 eligible publications, and analyzed co-occurrence via VOSviewer. As shown in Figure 8A, 128 keywords with more than 15 co-occurrences were subject to the mapping analysis, which yielded 3 distinct clusters: cluster 1 (mechanism-related research, red), cluster 2 (clinical research, green), and cluster 3 (diagnostic research, blue). The size of each keyword indicated its frequency of co-occurrence. In cluster 1, relevant keywords included effect (191 times), role (145 times), cell (97 times), expression (88 times) and increase (83 times). In cluster 2, frequently appearing keywords were score (171 times), intensive care unit year (135 times), age (93 times), year (93 times) and admission (90 times). In cluster 3, the main keywords were hospital (95 times), diagnosis (87 times), child (72 times), sample (66 times) and assay (51 times). Supplemental Table 1 shows the detailed results of all 128 keywords.

In Figure 8B, we colored all keywords according to the time when the word appeared. The color of keywords represented their appearing time, with the color blue indicating early appearance and the color yellow recent emergence. As for 2001–2011, zymosan (cluster 1, keyword AAY 2007.5) peritonitis (cluster 1, keyword AAY 2007.5) and animal (cluster 1, keyword AAY 2007.9) were the main research topics. In addition, analysis of the hot vocabulary of cluster 1 revealed “oxidative stress” (cluster 1, AAY 2013.4), blood urea nitrogen (AAY 2012.7) and pathway (AAY 2012.2) might be the research hotspots. In cluster 2 (clinical research), the latest hot word was “area” (AAY 2014.3), appearing 47 times. In cluster 3 (diagnostic Science Research), “detection” (AAY 2013.7) and “performance” (AAY 2013.7) were considered as the novel keywords, which appeared 24 and 21 times, respectively.

## Discussion

### Research Trends in MODS

It could be clearly seen from Figure 2 that 2009 and 2019 were the 2 years with the highest volume of MODS-related publications. The search of MODS-related literature published in 2009 and 2019 revealed that many international conferences on critical illness, war trauma and microbiology were held in these 2 years. These conferences greatly stimulated the publication of MODS-related articles. Through further exploration, we found that the article “The cytokine storm and factors determining the sequence and severity of organ dysfunction in multiple organ dysfunction syndrome” published in 2008 innovatively proposed that inflammatory factor storm was closely related to MODS, which might be one of its important pathogeneses (25). This conclusion undoubtedly provided a huge impetus for the related research of MODS. Of note, “The Third International Consensus on Sepsis and Septic Shock (Sepsis-3)” defined sepsis as a life-threatening organ dysfunction caused by a dysregulated host response to infection, reflecting that MODS was the terminal stage of sepsis (26). This new definition of sepsis triggered a climax of MODS research. Given the periodicity of the publications, it was not difficult to understand the blowout of MODS-related articles in 2019.

The United States, Germany, and the United Kingdom ranked the top three in terms of total citations and H-index in the MODS field. The United States made the most outstanding contribution to the development of global MODS research, as evidenced by the number of published articles, the frequency of citations and the H-index. The concept of MODS was first proposed by American and European scholars, indicating that the United States paid attention to this issue earlier than most countries in the world. In addition, the conditions for basic medical research and clinical trials in the United States seemed to be superior, characterized by advanced equipment, professional researchers, and sufficient funds. Moreover, the United States has some high-impact journals, excellent institutions and prolific authors related to MODS, and numerous high-level and influential international conferences were held in the United States. All of these advantages made the United States a leader in this field.

It was worth noting that although China ranked second in the total number of publications, it ranked only fifth and seventh in terms of citation frequency and H-index. There may be many reasons for the imbalance between the quantity and quality of Chinese publications. First of all, Chinese researchers first published an article in the related fields in 1992, but the number of annually-published articles was relatively small before 2009 (27). Therefore, it will take longer for China's citation frequency to catch up with that of other countries. Second, the diagnosis and treatment of MODS lacks standardization in China. In most Chinese hospitals, even in tertiary hospitals, medical staff do not regularly perform MODS-related scoring on critically ill patients, resulting in a high missed diagnosis rate for MODS patients. Third, China lacks high-quality multi-center randomized clinical trials (RCT) to provide reliable evidence for clinical practice. Additionally, compared with developed countries in Europe and the United States, the medical infrastructure and health system in China were still relatively backward, which to some extent limited the advance of basic and clinical MODS-related research in China. Finally, some high-quality literatures published in non-English journals may also be part of the reasons.

It can be seen from the time curve that since 2009, the number of articles published by China grew rapidly. Regrettably, although China has ranked second in the world in the total number of published articles in the past 20 years, it lags behind Germany, the United Kingdom and Canada in terms of the citation frequency and H-index. In addition, H-index of Italy and the Netherlands was comparable to that of China, although the number of publications by these two countries ranked only the 6th and 8th, respectively. Thus, it is urgent to improve the quality of scientific research papers in China.

Among the top 10 institutions in the MODS field, the United States boasted seven, demonstrating its absolute dominance in this field. The top three institutions that published the most articles in this field were the University of Pittsburgh, COLORADO University, and Denver HLTH MED CTR, which are all in the United States. These results indicate that the United States has the most elite institutions and also explain why this country maintains a leading position in the MODS field. There were three other universities on the top 10 list, namely the University of Messina in Italy, the University of Halle-Wittenberg in Germany, and the University of Cayetano in Peru. However, the imbalanced distribution of top institutions means we are still far from reaching the goal of “scientific research without borders,” suggesting that more elite institutions outside the United States should involve in MODS-related research to provide more impetus for basic and clinical research.

As to the journals, *Critical Care Medicine* published 106 papers in the field of MODS, far ahead of other journals. *Shock* and *Intensive Care Medicine* were the other major journals publishing MODS-related articles. This indicates that the focus and hotspots of future advances in this field may appear in the above-mentioned journals.

In terms of authors, Moore EE and Carcillo JA from the United States and Cuzzocrea S from Italy published the most articles related to MODS. Moore EE and Carcillo JA mainly explored the dysfunction of regulatory T cells and macrophages in the pathogenesis of sepsis, while Cuzzocrea S focused on the potential role of neutrophils in sepsis and attempted to regulate the functions of neutrophils to reduce the pathological changes of sepsis. Although Cuzzocrea S from University of Messina ranked second in the total number of published papers, he ranked only seventh in terms of citation frequency, which may be related to the late publication of his papers. It was worth noting that the institutions of the top 10 highly productive authors were relatively concentrated. To be specific, the four professors in Italy were all from the University of Messina; the three professors in the United States except Sauaia A were all from the University of Pittsburgh; the two professors in Germany were both from Martin Luther University. In addition, cooperation between the authors is of great significance to the research of MODS. For example, Moore E. E. was listed as a co-author in many papers of the above-mentioned authors, indicating that he has close cooperative relations with authors from different institutions. We believe that these researchers may play a unique and indispensable role in this field. Their research not only have a wide-ranging impact on the past development of the field, but also point out the direction of the hotspots and future development in this field.

### Research Focuses on MODS

The most cited papers have great academic influence in the field. The details of the top 10 cited in the MODS field are shown in Table 1. The paper “Immunosuppression in Patients Who Die of Sepsis and Multiple Organ Failure” has been cited 884 times since its publication and is the most cited paper in MODS fields. This research was published in *JAMA* in 2011, and the corresponding authors were Hotchkiss and Richard S. They put forward for the first time that the biochemical, flow cytometry, and immunohistochemical results of sepsis patients who died in the ICU were consistent with those of sepsis patients died from immunosuppression. This study was the first to confirm the existence of an immunosuppressive state at the onset of sepsis. They groundbreakingly proposed that targeted immune-enhancement therapy for sepsis patients may be an effective method (24). The second and third highly cited articles studied the important role of endothelial cells and neutrophils in the pathogenesis of MODS, which were published in *BLOOD* and *LANCET*, respectively (15, 28). Both articles focused on the pathogenesis of MODS, emphasizing that immunosuppression caused by cellular dysfunction was the main abnormality in MODS patients. Timely improvement of the endothelial cell and immune cell dysfunction and accurate immunotherapy would possibly show important clinical significance in the pathogenesis of MODS. In fact, most of the top 10 cited papers are directed at the pathogenesis of MODS. The relationship between inflammation, immunity and the pathogenesis of MODS has always been the focus of research (14, 25, 29). Early elucidations of these mechanisms will hopefully promote therapeutic advances and help to reduce the mortality of MODS patients, although current ongoing or completed clinical studies of MODS are mainly focused on immune-related drugs (NCT03769844, 01186783, 03119701, and 03518203).

The most recent hotspot was the “survival curve” (cluster 2, AAY is 2016.7). In fact, 2 of the 5 newly emerged words were from the “clinical research” group, namely “Area Under the Curve” and “Survival Curve.” As shown in the Figure 8, the “clinical research” cluster received slightly less attention than the “mechanism-related research” cluster. However, the former contained some new words that appeared in recent years, indicating that the research focus of MODS has gradually shifted from mechanism research to clinical research. Hence how to improve the survival rate of MODS patients has become the focus of relevant scholars. According to the cluster relationship diagram formed by keywords, we can clearly see that clinical research cluster was closely related to “diagnostic research” cluster, whereas “clinical research” and “mechanism-related research” clusters were relatively less connected, indicating that the conversion speed of basic research and clinical research in the field of MODS needs to be accelerated. In terms of mechanism research, a relatively new keyword was “oxidative stress,” which is a negative effect produced by free radicals in the body and is considered to be an important factor leading to aging and disease (30). The appearance of this term in “clinical research” cluster also shows that the relationship between oxidative stress and poor prognosis of MODS has become a relatively new hotspot (31). It is worth mentioning that “China” is a new keyword in the “diagnostics and laboratory research” cluster. On the one hand, the research of Chinese scholars in MODS-related fields has increased, with a growing number of publications in China in recent years. On the other hand, COVID-19 suddenly broke out in Wuhan, China at the end of 2019, and most of the patients with severe illness progressed to MODS in the later stage (32, 33). These are major reasons why “China” became a hot keyword.

In general, through the analysis of the status quo and focus of MODS research, we hope to give some novel inspiration to relevant researchers. For example, in terms of basic research, the role of oxidative stress in the onset and deterioration of MODS is a current research hotspot. Mitochondrial metabolism and mitophagy are closely related to oxidative stress, thereby may mitochondrial metabolism and mitophagy be an innovative direction in the field of MODS? Currently, MODS-related clinical studies are mainly focused on survival and prognosis researches, the exploration of biomarkers that can predict poor outcomes in MODS patients and timely intervention are the priority of current and future research. In MODS diagnosis-related research, how to more accurately predict the occurrence of MODS in patients with COVID-19 is crucial to reducing the mortality rate of COVID-19 patients.

### Strengths and Limitations

The MODS-related papers in this study were extracted from the core database of WOS, which is an extension of the scientific citation index. We comprehensively and objectively summarized the development status of MODS in the past 20 years from the perspective of bibliometrics and predicted and analyzed the research hotspots of MODS, which we believe will provide some reference for scholars in related fields to carry out researches. In addition, all searches were completed in 1 day, thus avoiding the deviation resulting from database updates. We assume that these latest publications may not be cited frequently in a short term, thus will not affect our conclusions. Nevertheless, some limitations are inevitable. First, due to our inclusion criteria, we only extracted English publications. Therefore, important researches in non-English language were ignored and excluded from the analysis, we tried to search these 55 non-English articles and found that these articles were mainly Russian or Latin ones, and conducting a separate analysis of these 55 non-English articles might be more reasonable. Second, we limited the types of articles to articles and reviews. Other influential work published in the form of conference abstracts or letters were also ignored. Third, we only included research papers published from 2001 to July 31, 2021, which means that no keywords before 2001 were included. Forth, in order to facilitate the analysis, we only searched the WOS core databases, and the MODS-related articles from the non-core and other database were ignored, which may also have a certain impact on our results. Finally, we mainly reflected the quality of publications from the perspective of citation frequency and H-index, and it is better to evaluate the quality of publications from a multi-dimensional perspective.

## Conclusions

To sum up, this study summarizes global research status of MODS during 2001–2020. The United States made the greatest contribution in this important field. China, published the second largest number of papers, but the quality of these papers does need to be improved. The latest research and advances can be found in *Critical Care Medicine, Shock*, and *Intensive Care Medicine*. Moore EE, Cuzzocrea S, and Carcillo JA are academic leaders and have the most academic influence in this field. Basic research in MODS-related fields developed rapidly and received sufficient attention from researchers in the early stage. However, in recent years, the research hotspot of MODS has gradually transitioned from basic research to clinical research. The proportion of clinical research on MODS has gradually increased, but the transformation of basic mechanism research into clinical application still has a long way to go. We hope that this research could provide MODS-related researchers with a clear understanding of the current status and trends of MODS, and encourage more investigators worldwide to participate in this field for the sake of reducing the mortality rate of MODS patients.

## Data Availability Statement

The original contributions presented in the study are included in the article/Supplementary Material, further inquiries can be directed to the corresponding author/s.

## Author Contributions

X-hD and R-qY conceptualized, supervised, and edited the manuscript. P-yZ, YX, and Z-bT extracted all data and performed the bibliometric analyses. ZM, S-yL, and X-pY undertook and refined the searches. P-yZ and YX co-drafted the paper. All authors contributed to and revised the final manuscript.

## Funding

This work was supported by grants from the National Natural Science Foundation of China (Nos. 81801935 and 81871317) and the Key Project of Military Medical Innovation Program (Nos. 18CXZ025 and 18CXZ026).

## Conflict of Interest

The authors declare that the research was conducted in the absence of any commercial or financial relationships that could be construed as a potential conflict of interest.

## Publisher's Note

All claims expressed in this article are solely those of the authors and do not necessarily represent those of their affiliated organizations, or those of the publisher, the editors and the reviewers. Any product that may be evaluated in this article, or claim that may be made by its manufacturer, is not guaranteed or endorsed by the publisher.

## Supplementary Material

Supplementary Figure 1 The analysis of countries/regions in publications of MODS. **(A)** The cooperation of countries/regions in MODS, the circle with a large size represented the countries/regions that published more articles; **(B)** Distribution of countries/regions was presented according to the appearance for the average time.

Supplementary Figure 2 The network map of productive authors engaging in MODS. **(A)** The circle with a large size represented the author that published more articles; **(B)** Distribution of authors was presented according to the appearance for the average time.

Supplementary Table 1 The analytic consequence of 128 keywords with at least 15 occurrence times.

## Abbreviations

MODS: Multiple Organ Dysfunction Syndrome
ICU: intensive care unit
PLA: People's Liberation Army
DC: dendritic cells
WOS: Web of Science
SCI-E: Science Citation Index-Expanded
TI: title
SIRS: systematic inflammatory response syndrome
AAY: average appearing year
IF: impact factor
JCR: Journal Citation Reports
JAMA: The Journal of the American Medical Association
SOFA: sequential organ failure assessment
APACHE II: acute physiology and chronic health evaluation II
RCTs: Randomized Clinical Trials.
