# Supplementary figures and images for: Global Research Status of Multiple Organ Dysfunction Syndrome During 2001–2021: A 20-Year Bibliometric Analysis
Source: Front Med (Lausanne). 2022 Mar 4;9:814381. doi: 10.3389/fmed.2022.814381 (PMC8931214; doi:10.3389/fmed.2022.814381)

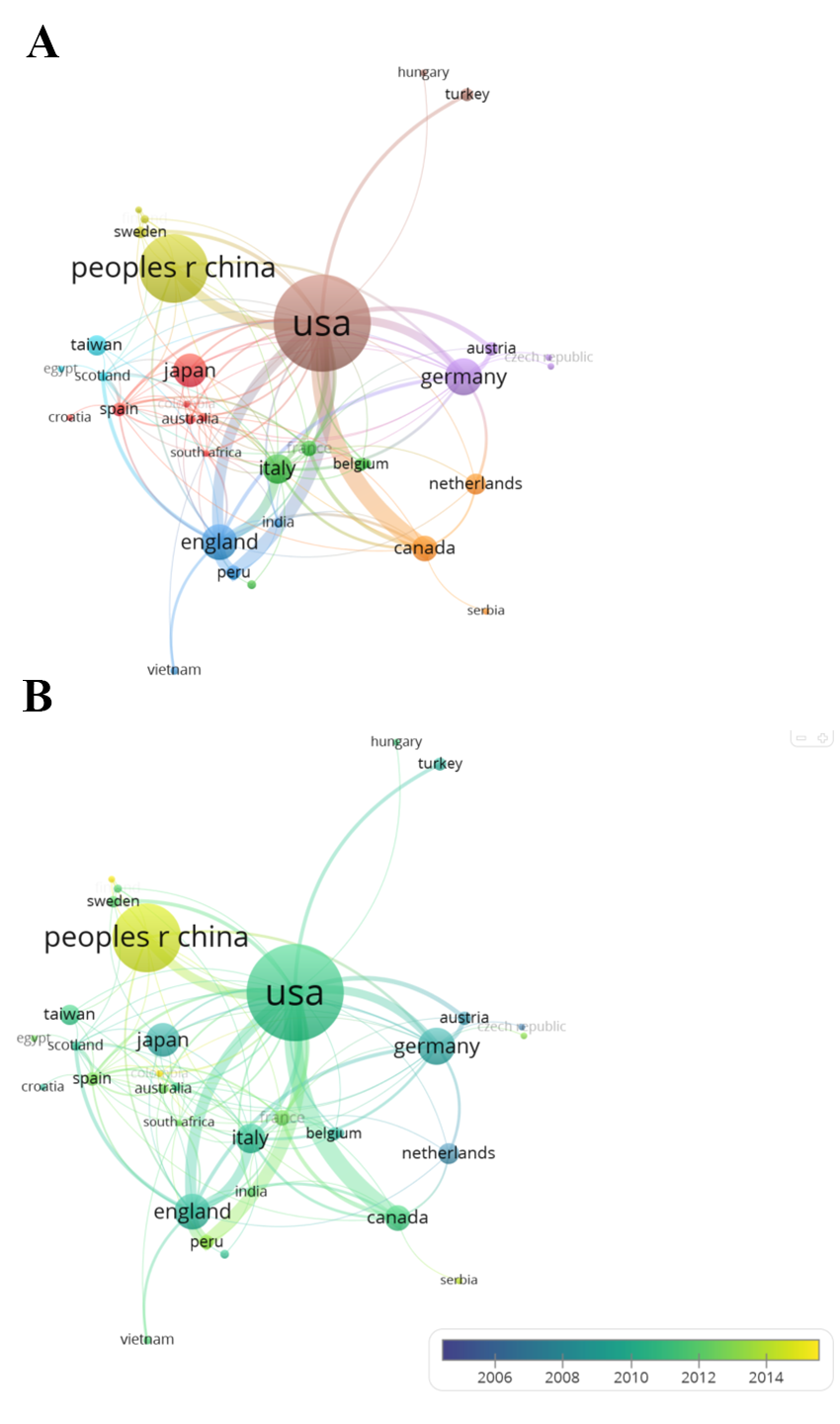

Supplement: Supplementary Figure 1 — The analysis of countries/regions in publications of MODS. (A) The cooperation of countries/regions in MODS, the circle with a large size represented the countries/regions that published more articles; (B) Distribution of countries/regions was presented according to the appearance for the average time. [file Image_1.TIF]

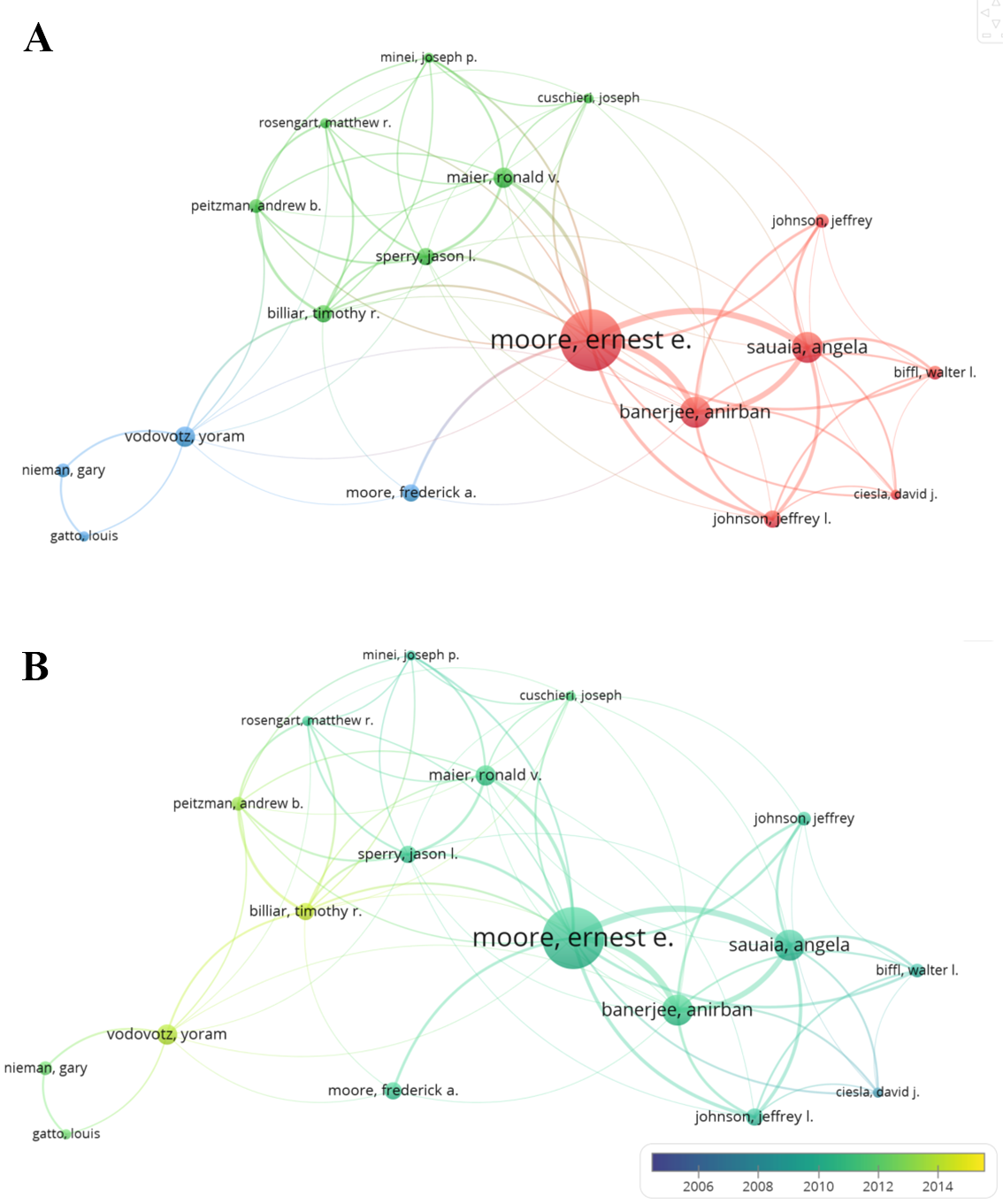

Supplement: Supplementary Figure 2 — The network map of productive authors engaging in MODS. (A) The circle with a large size represented the author that published more articles; (B) Distribution of authors was presented according to the appearance for the average time. [file Image_2.TIF]
