# Supplementary material for: Global Research Status of Multiple Organ Dysfunction Syndrome During 2001–2021: A 20-Year Bibliometric Analysis
Source: Front Med (Lausanne). 2022 Mar 4;9:814381. doi: 10.3389/fmed.2022.814381 (PMC8931214; doi:10.3389/fmed.2022.814381)
Supplement: Supplementary Table 1 — The analytic consequence of 128 keywords with at least 15 occurrence times. [file Table_1.DOC]

| Rank | Keywords | Cluster | Links | Occurrences | Average appearing years (AAY) | Average citations |
| --- | --- | --- | --- | --- | --- | --- |
| 1 | absence | 1 | 75 | 19 | 2009.8 | 25.0526 |
| 2 | activation | 1 | 106 | 58 | 2011.4 | 44.7241 |
| 3 | activity | 1 | 100 | 62 | 2009.0 | 26.1774 |
| 4 | acute kidney injury | 3 | 71 | 19 | 2015.3 | 16.4211 |
| 5 | acute physiology | 2 | 77 | 32 | 2012.1 | 19.1875 |
| 6 | administration | 1 | 107 | 64 | 2009.0 | 29.0781 |
| 7 | admission | 2 | 106 | 90 | 2012.5 | 30.7889 |
| 8 | age | 2 | 112 | 93 | 2011.6 | 54.1613 |
| 9 | animal | 1 | 83 | 46 | 2007.9 | 31.1739 |
| 10 | apache | 2 | 66 | 19 | 2009.9 | 29.6316 |
| 11 | apache ii | 2 | 66 | 20 | 2013.2 | 14.8 |
| 12 | apache ii score | 2 | 80 | 35 | 2011.7 | 23 |
| 13 | apoptosis | 1 | 75 | 22 | 2009.5 | 32.8636 |
| 14 | area | 2 | 96 | 47 | 2014.3 | 25.2766 |
| 15 | article | 3 | 49 | 29 | 2009.7 | 74.1724 |
| 16 | assay | 3 | 94 | 51 | 2012.5 | 15.549 |
| 17 | beneficial effect | 1 | 70 | 16 | 2008.4 | 26 |
| 18 | beta | 1 | 88 | 29 | 2012.4 | 25.6207 |
| 19 | blood urea nitrogen | 1 | 79 | 17 | 2012.7 | 18 |
| 20 | body weight | 1 | 74 | 26 | 2008.8 | 15.1538 |
| 21 | cell | 1 | 111 | 97 | 2010.9 | 34.5773 |
| 22 | child | 3 | 90 | 72 | 2010.6 | 23.7083 |
| 23 | chronic health evaluation | 2 | 66 | 18 | 2008.9 | 34.8333 |
| 24 | comparison | 2 | 98 | 38 | 2009.7 | 22.3947 |
| 25 | confidence interval | 2 | 65 | 20 | 2013.0 | 47.4 |
| 26 | control group | 1 | 94 | 46 | 2011.8 | 15.913 |
| 27 | criterium | 2 | 105 | 55 | 2010.4 | 35.4545 |
| 28 | cytokine | 1 | 116 | 64 | 2009.3 | 48.2188 |
| 29 | damage | 1 | 103 | 78 | 2010.0 | 34.6795 |
| 30 | day mortality | 2 | 59 | 17 | 2012.9 | 35.3529 |
| 31 | december | 2 | 66 | 16 | 2012.4 | 30.25 |
| 32 | degree | 1 | 98 | 35 | 2007.7 | 30.5714 |
| 33 | detection | 3 | 51 | 24 | 2013.7 | 27.9583 |
| 34 | diagnosis | 3 | 101 | 87 | 2012.5 | 14.1264 |
| 35 | disseminated intravascular coagulation | 3 | 64 | 18 | 2010.8 | 25.2222 |
| 36 | dose | 1 | 94 | 40 | 2009.8 | 21.175 |
| 37 | duration | 2 | 86 | 28 | 2012.0 | 56.7143 |
| 38 | effect | 1 | 122 | 196 | 2010.4 | 25.8622 |
| 39 | endotoxemia | 1 | 72 | 19 | 2010.3 | 26.6316 |
| 40 | endotoxin | 1 | 70 | 23 | 2008.7 | 29.087 |
| 41 | epidemiology | 2 | 60 | 20 | 2010.8 | 59.7 |
| 42 | evaluation | 3 | 87 | 44 | 2011.2 | 19.9091 |
| 43 | expression | 1 | 111 | 88 | 2011.8 | 29.1932 |
| 44 | field | 3 | 44 | 21 | 2013.4 | 10.5714 |
| 45 | heart | 1 | 92 | 29 | 2011.2 | 16.7931 |
| 46 | hospital | 3 | 114 | 95 | 2011.6 | 31.9368 |
| 47 | hospital mortality | 2 | 53 | 15 | 2012.6 | 28.4 |
| 48 | hospitalization | 3 | 60 | 19 | 2014.0 | 20.8947 |
| 49 | icu | 2 | 98 | 62 | 2010.3 | 48.3387 |
| 50 | icu admission | 2 | 62 | 17 | 2012.5 | 43.2941 |
| 51 | ill child | 3 | 64 | 23 | 2012.8 | 45 |
| 52 | incidence | 2 | 99 | 59 | 2010.2 | 47.1356 |
| 53 | increase | 1 | 121 | 83 | 2008.5 | 33.2651 |
| 54 | injury severity score | 2 | 79 | 36 | 2012.1 | 42.5833 |
| 55 | intensive care unit | 2 | 120 | 135 | 2009.8 | 53.5111 |
| 56 | intestine | 1 | 56 | 22 | 2008.5 | 17.4091 |
| 57 | intravascular coagulation | 3 | 67 | 22 | 2009.9 | 27.7273 |
| 58 | iss | 2 | 71 | 28 | 2013.9 | 41.5714 |
| 59 | january | 2 | 81 | 26 | 2013.3 | 23.4231 |
| 60 | kidney | 1 | 96 | 49 | 2010.9 | 21.7755 |
| 61 | length | 2 | 82 | 28 | 2011.6 | 33.3214 |
| 62 | life | 3 | 79 | 36 | 2012.2 | 22.3889 |
| 63 | lipopolysaccharide | 1 | 84 | 30 | 2009.3 | 63.9 |
| 64 | literature review | 3 | 43 | 18 | 2016.2 | 17.1111 |
| 65 | liver | 1 | 93 | 64 | 2010.8 | 19.9531 |
| 66 | loss | 1 | 71 | 27 | 2009.4 | 55.1852 |
| 67 | lps | 1 | 69 | 15 | 2011.1 | 29.5333 |
| 68 | lung | 1 | 102 | 74 | 2010.0 | 39.2162 |
| 69 | mg kg | 1 | 62 | 30 | 2007.4 | 23.0333 |
| 70 | microscopic observation drug susceptibility | 3 | 16 | 20 | 2012.0 | 11.25 |
| 71 | min | 1 | 85 | 33 | 2008.4 | 23.1515 |
| 72 | month | 3 | 75 | 36 | 2012.8 | 15.9444 |
| 73 | mouse | 1 | 70 | 53 | 2010.0 | 23.3962 |
| 74 | need | 3 | 69 | 20 | 2012.1 | 21.25 |
| 75 | nitric oxide | 1 | 72 | 18 | 2009.2 | 21.4444 |
| 76 | none | 2 | 70 | 22 | 2006.6 | 55.9091 |
| 77 | nonsurvivor | 2 | 63 | 15 | 2008.7 | 28.3333 |
| 78 | odds ratio | 2 | 71 | 19 | 2013.2 | 41.3684 |
| 79 | organ injury | 1 | 80 | 28 | 2009.5 | 43.3214 |
| 80 | oxidative stress | 1 | 77 | 22 | 2013.4 | 23.9545 |
| 81 | pathogenesis | 1 | 100 | 64 | 2010.0 | 44.4844 |
| 82 | pathway | 1 | 86 | 41 | 2012.2 | 19.9024 |
| 83 | performance | 3 | 59 | 21 | 2013.7 | 22.5238 |
| 84 | peritonitis | 1 | 82 | 22 | 2007.5 | 25.0455 |
| 85 | picu | 3 | 63 | 20 | 2014.6 | 24.2 |
| 86 | plasma | 1 | 94 | 35 | 2008.3 | 43.6571 |
| 87 | plasma exchange | 3 | 44 | 25 | 2010.4 | 22.8 |
| 88 | plasma level | 1 | 82 | 19 | 2008.2 | 31.1579 |
| 89 | postinjury multiple organ failure | 2 | 63 | 22 | 2007.5 | 59.8636 |
| 90 | prediction | 2 | 79 | 28 | 2011.7 | 35.6786 |
| 91 | predictor | 2 | 94 | 50 | 2011.8 | 43.12 |
| 92 | present study | 1 | 99 | 35 | 2011.6 | 15.3143 |
| 93 | presentation | 3 | 72 | 27 | 2012.3 | 11.963 |
| 94 | production | 1 | 102 | 53 | 2008.8 | 35.434 |
| 95 | protective effect | 1 | 78 | 23 | 2011.6 | 23.3478 |
| 96 | range | 3 | 93 | 39 | 2012.6 | 42.4103 |
| 97 | rat | 1 | 78 | 73 | 2009.7 | 16.5205 |
| 98 | rat model | 1 | 58 | 16 | 2011.8 | 13.75 |
| 99 | receptor | 1 | 92 | 37 | 2011.8 | 43.9459 |
| 100 | release | 1 | 85 | 33 | 2009.0 | 40.4242 |
| 101 | renal dysfunction | 1 | 80 | 19 | 2007.9 | 20.8947 |
| 102 | report | 3 | 74 | 56 | 2012.1 | 10.0536 |
| 103 | risk factor | 2 | 91 | 51 | 2011.7 | 45.8824 |
| 104 | role | 1 | 123 | 145 | 2010.4 | 35.8897 |
| 105 | saline | 1 | 62 | 27 | 2007.7 | 17.3704 |
| 106 | score | 2 | 122 | 171 | 2011.3 | 31.9766 |
| 107 | sensitivity | 3 | 77 | 39 | 2012.5 | 24.0256 |
| 108 | sequential organ failure assessment | 2 | 90 | 32 | 2011.1 | 33.75 |
| 109 | significant increase | 1 | 84 | 17 | 2009.6 | 23.2353 |
| 110 | sofa | 2 | 83 | 34 | 2010.8 | 27.7941 |
| 111 | sofa score | 2 | 77 | 31 | 2012.0 | 30.5806 |
| 112 | specificity | 3 | 72 | 36 | 2013.4 | 21.0278 |
| 113 | stay | 2 | 95 | 40 | 2011.2 | 32.575 |
| 114 | study period | 2 | 70 | 17 | 2009.4 | 56.4706 |
| 115 | survival rate | 1 | 95 | 28 | 2010.6 | 36.0714 |
| 116 | survivor | 2 | 96 | 38 | 2011.7 | 25.6053 |
| 117 | systemic inflammation | 1 | 83 | 28 | 2010.0 | 40.9643 |
| 118 | thrombocytopenia | 3 | 61 | 28 | 2010.5 | 18.8571 |
| 119 | tissue | 1 | 83 | 47 | 2011.0 | 27.0426 |
| 120 | tnf | 1 | 71 | 16 | 2010.4 | 26.5 |
| 121 | tnf alpha | 1 | 97 | 44 | 2011.5 | 28.1591 |
| 122 | trauma patient | 2 | 92 | 56 | 2011.6 | 34.4821 |
| 123 | tuberculosis | 3 | 24 | 31 | 2012.5 | 12.1935 |
| 124 | tumor necrosis factor alpha | 1 | 85 | 27 | 2010.6 | 29.4074 |
| 125 | understanding | 3 | 64 | 29 | 2011.3 | 69.0345 |
| 126 | vitro | 1 | 60 | 15 | 2011.8 | 19.2 |
| 127 | year | 2 | 117 | 93 | 2011.8 | 43.4086 |
| 128 | zymosan | 1 | 60 | 40 | 2007.5 | 18.75 |

**Table S1** The analytic consequence of 128 keywords with at least 15 occurrence times
